# Disruption of postnatal folliculogenesis and development of ovarian tumor in a mouse model with aberrant transforming growth factor beta signaling

**DOI:** 10.1186/s12958-017-0312-z

**Published:** 2017-12-08

**Authors:** Yang Gao, Xin Fang, David F. Vincent, David W. Threadgill, Laurent Bartholin, Qinglei Li

**Affiliations:** 1 Department of Veterinary Integrative Biosciences, College of Veterinary Medicine and Biomedical Sciences, Texas A&M University, College Station, TX 77843 USA; 2 Department of Veterinary Pathobiology, College of Veterinary Medicine and Biomedical Sciences, Texas A&M University, College Station, TX 77843 USA; 3 Department of Molecular and Cellular Medicine, Texas A&M Health Science Center, Texas A&M University, College Station, TX 77843 USA; 4 Centre de Recherche en Cancérologie de Lyon, INSERM U1052, CNRS UMR5286, Université Lyon 1, Centre Léon Bérard, F-69000 Lyon, France; 5 Cancer Research UK Beatson Institute, Garscube Estate, G61 1BD, Glasgow, UK

**Keywords:** TGFB, Ovary, *Gdf9*-iCre, Tumor

## Abstract

**Background:**

Transforming growth factor beta (TGFB) superfamily signaling is implicated in the development of sex cord-stromal tumors, a category of poorly defined gonadal tumors. The aim of this study was to determine potential effects of dysregulated TGFB signaling in the ovary using Cre recombinase driven by growth differentiation factor 9 (*Gdf9*) promoter known to be expressed in oocytes.

**Methods:**

A mouse model containing constitutively active TGFBR1 (TGFBR1^CA^) using *Gdf9*-iCre (termed TGFBR1-CA^G9Cre^) was generated. Hematoxylin and eosin (H & E) staining, follicle counting, and immunohistochemistry and immunofluorescence analyses using antibodies directed to Ki67, forkhead box L2 (FOXL2), forkhead box O1 (FOXO1), inhibin alpha (INHA), and SRY (sex determining region Y)-box 9 were performed to determine the characteristics of the TGFBR1-CA^G9Cre^ ovary. Terminal deoxynucleotidyl transferase (TdT) labeling of 3’-OH ends of DNA fragments, real-time PCR, and western blotting were used to examine apoptosis, select gene expression, and TGFBR1 activation. RNAscope in situ hybridization was used to localize the expression of GLI-Kruppel family member GLI1 (*Gli1*) in ovarian tumor tissues.

**Results:**

TGFBR1-CA^G9Cre^ females were sterile. Sustained activation of TGFBR1 led to altered granulosa cell proliferation evidenced by high expression of Ki67. At an early age, these mice demonstrated follicular defects and development of ovarian granulosa cell tumors, which were immunoreactive for granulosa cell markers including FOXL2, FOXO1, and INHA. Further histochemical and molecular analyses provided evidence of overactivation of TGFBR1 in the granulosa cell compartment during ovarian pathogenesis in TGFBR1-CA^G9Cre^ mice, along with upregulation of *Gli1* and *Gli2* and downregulation of *Tgfbr3* in ovarian tumor tissues.

**Conclusions:**

These results reinforce the role of constitutively active TGFBR1 in promoting ovarian tumorigenesis in mice. The mouse model created in this study may be further exploited to define the cellular and molecular mechanisms of TGFB/activin downstream signaling in granulosa cell tumor development. Future studies are needed to test whether activation of TGFB/activin signaling contributes to the development of human granulosa cell tumors.

**Electronic supplementary material:**

The online version of this article (10.1186/s12958-017-0312-z) contains supplementary material, which is available to authorized users.

## Background

Transforming growth factor beta (TGFB) superfamily members regulate a variety of biological processes in a contextually dependent manner via the interaction with membrane associated TGFBR1/TGFBR2 serine/threonine kinase receptor complexes and downstream SMAD proteins [1]. A growing body of research documents critical roles of TGFB signaling in reproductive development, function, and dysfunction [2, 3]. TGFB signaling is known for its tumor suppressive function, particularly in epithelial cancer cells [4, 5]. However, TGFB signaling also promotes cancer progression through modulating tumor cell invasion and metastasis [6]. Recently, we have shown that TGFB pathway activation plays a crucial role in pancreatic tumor initiation, through its capacity to induce Acinar-to-Ductal metaplasia, providing a favourable environment for KRAS^G12D^-dependent carcinogenesis [7].

Disruption of genes encoding TGFB superfamily signaling mediators [e.g., inhibin alpha (*Inha*), bone morphogenetic protein (BMP)-responsive SMADs (i.e., *Smad1/5*), and BMP type 1 receptors (i.e., *Bmpr1a* and *Bmpr1b*)] results in the development of sex cord-stromal tumors [8–10]. Studies have revealed tumorigenic function of activin-responsive SMAD3 and tumor suppressive roles of inhibins and BMPs in the gonad [8–11]. A cornerstone study has demonstrated that nearly all adult granulosa cell tumors (GCTs), a major subtype of sex cord-stromal tumors, harbor a somatic mutation of forkhead box L2 (*FOXL2*; 402C→G; C134W) [12]. However, the specific role of *FOXL2* mutation and its associated tumorigenic signals in GCTs remain to be clarified. Therefore, it is imperative to define the mechanism underlying the initiation and progression of ovarian GCTs, with an ultimate goal of developing new therapeutic strategies for this type of poorly characterized tumor.

As the functional unit of the ovary, a follicle consists of granulosa and theca somatic cells and a germ cell, the oocyte. Through secretion of paracrine growth factors, the oocyte regulates multiple functions of granulosa cells including, but not limited to, apoptosis, proliferation and differentiation, steroidogenesis, and metabolism [13, 14]. It is noteworthy that dysregulation of phosphoinositide-3-kinase (PI3K)-AKT signaling in the mouse oocyte impairs normal ovarian function and causes ovarian GCT formation [15]. In an early report, we have shown that constitutive activation of TGFB signaling in mouse granulosa cells promotes ovarian tumorigenesis [16]. To further explore the tumorigenic function of dysregulated TGFB signaling in distinct cellular compartments of the ovary, we created a mouse model that harbors constitutively active TGFBR1 using growth differentiation factor 9 (*Gdf9*)-iCre known to be expressed in the oocyte.

## Methods

### Animals

Experimental protocols using mice were approved by the Institutional Animal Care and Use Committee at Texas A&M University. Mice were on a C57/129 mixed background. The *Gdf9-*iCre mice have been used to delete genes in oocytes from the primordial follicle stage [17, 18]. Zona pellucida glycoprotein 3 (*Zp3*)-Cre mice (stock no. 003651) [19] and *Gt(ROSA)26Sor* ^*tm1Sor*^ */J* mice (stock no. 003474) containing a Cre-inducible *LacZ* allele were purchased from the Jackson Laboratory [20]. Mice carrying a latent constitutively active TGFBR1 (*TGFBR1* ^CA^) that is knocked into the hypoxanthine guanine phosphoribosyl transferase (*Hprt*) locus were generated previously [21, 22]. *TGFBR1* ^CA flox/+^ male mice were crossed with *Gdf9*-iCre female mice to generate *TGFBR1* ^CA flox/+^; *Gdf9*-iCre mice (TGFBR1-CA^G9Cre^; experimental group) and *TGFBR1* ^CA flox/+^ mice (control group; Ctrl). Genotypes of mice were determined by genomic PCR using tail DNA and primers described elsewhere [17, 21].

### Histology and follicle counting

Hematoxylin and eosin (H & E) staining was conducted using standard procedures. To perform follicle counting, ovaries were first serially sectioned (5 μm) and stained with periodic acid Schiff’s (PAS) and hematoxylin. Follicles were counted from every 5th section. Follicle classification was based on previously established criteria [23, 24]. Primordial and primary follicles were counted, regardless of the status of the oocyte nucleus. For secondary follicles, only those with visible oocyte nucleus in the section were counted as previously described [23]. Since the oocyte diameter for primordial and primary follicles averages ~14 μm, the same follicle could be counted every two sections. Hence, the estimated follicle numbers per ovary were calculated by multiplying the cumulative number of each ovary by 5 and then dividing by 2 as described [23].

### Immunohistochemistry and Immunofluorescence

Immunohistochemistry was performed using an avidin-biotin complex (ABC) kit purchased from Vector Laboratories [16, 25]. Briefly, sections were deparaffinized and rehydrated prior to antigen retrieval that was performed by boiling the sections in acidic sodium citrate buffer (pH 6.0) using a microwave. Following H_2_O_2_ treatment and blocking, the sections were incubated with primary antibodies directed to FOXL2 (Abcam; ab5096; 1:1500), INHA (AbD Serotec; MCA951ST; 1:300), forkhead box O1 (FOXO1) (Cell Signaling; 2880; 1:400), anti-Mullerian hormone (AMH) (Santa Cruz; sc-6886; 1:2000), and DEAD (Asp-Glu-Ala-Asp) box polypeptide 4 (DDX4) (Cell Signaling; 8761; 1:500). Subsequently, the sections were incubated with secondary antibodies and ABC reagents. Signals were developed using NovaRED Peroxidase Substrate Kit (Vector Laboratories). Coverslips were affixed to slides using Permount Mounting Medium (Fisher Scientific).

Immunofluorescence was performed using antibodies against DDX4 (Cell Signaling; 8761; 1:200), smooth muscle actin alpha (ACTA2) (Abcam; ab76549; 1:2000), and SRY (sex determining region Y)-box 9 (SOX9) (Millipore; AB5535; 1:1000) [16]. Secondary antibodies were conjugated with Alexa Fluor 594 or 488. Invitrogen ProLong Gold Slowfade media containing 4′,6-diamidino-2-phenylindole (DAPI) was used to mount the slides. Negative controls where primary antibodies were replaced by species and isotype-matched immunoglobulin Gs (IgGs) were included. Results were examined and recorded using an Olympus fluorescence microscope (IX47) with appropriate filter sets, equipped with a cooled CCD camera (XM10) and cellSens imaging software.

### In situ apoptosis analysis

A commercially available In Situ Apoptosis Detection Kit (Abcam) was used to detect apoptosis in ovarian samples from control and TGFBR1-CA^G9Cre^ mice at postnatal day 3 (PD3), PD5, PD7, PD12, and PD21. Briefly, paraffin sections were deparaffinized in xylene and rehydrated in a graded alcohol series before the treatment with Proteinase K. Then 3% H_2_O_2_ was added to inactivate endogenous peroxidases. Apoptotic cells were labeled with terminal deoxynucleotidyl transferase (TdT) that catalyzes the addition of biotin-labeled deoxynucleotides, followed by incubation with streptavidin-horseradish peroxidase (HRP) conjugate. Positive controls where tissue sections were treated with DNase I and negative controls where TdT was substituted with water were included. The signal was detected using 3,3′-diaminobenzidine (DAB) substrate and sections counterstained with Methyl Green.

### RNA preparation, reverse transcription, and real-time PCR

Total RNA was extracted from mouse ovaries using an RNeasy Mini Kit from Qiagen based on the manufacturer’s instructions. On-column DNase digestion was performed using RNase-free DNase Set (Qiagen). Total RNA was quantified prior to reverse transcription, where Superscript III and Oligo (dT)_12-18_ primers (Thermo Fisher Scientific) were used [25]. CFX Connect Real-time PCR Detection System (Bio-Rad) was used for gene expression analysis, with a 10 μl reaction volume containing cDNA, iTaq Universal SYBR Green Supermix (Bio-Rad), and gene specific primers [*Smad7*: 5′-GGGCTTTCAGATTCCCAACTT-3′ and 5′-CACGCGAGTCTTCTCCTCC-3′ [26]; *Inha*: 5′-CCTTTTGCTGTTGACCCTACG-3′ and 5′-AGGCATCTAGGAATAGAGCCTTC-3′ (primerbank ID 31982785a1); *Zp3*: 5′-ATGGCGTCAAGCTATTTCCTC-3′ and 5′-CGTGCCAAAAAGGTCTCTACT-3′ (primerbank ID 6756083a1); *Gli1*: 5′- CCAAGCCAACTTTATGTCAGGG-3′ and 5′-AGCCCGCTTCTTTGTTAATTTGA-3′ (primerBank ID 6754002a1); *Gli2*: 5′-CAACGCCTACTCTCCCAGAC-3′ and 5′-GAGCCTTGATGTACTGTACCAC-3′ (primerBank ID 21411092a1); and *Tgfbr3*: 5′- GGTGTGAACTGTCACCGATCA-3′ and 5′- GTTTAGGATGTGAACCTCCCTTG-3′ (PrimerBank ID 33469109a1)] [27]. Relative expression of target genes, normalized against ribosomal protein L19 (*Rpl19*), was calculated based on the ΔΔCT method [28].

### Western blotting

Ovarian protein samples prepared from control and TGFBR1-CA^G9Cre^ mice were subjected to western blotting analysis as described [16]. Briefly, total proteins were separated on 12% Tris gels and transferred to PVDF membranes (Bio-Rad), followed by incubation with the following primary antibodies at 4 °C overnight: pSMAD2 (Millipore; AB3849-I; 1:500 or Cell Signaling; 3101; 1:1000), SMAD2 (Cell Signaling; 5339; 1:1000), pSMAD3 (Abcam; ab52903; 1:1000), SMAD3 (Abcam; ab28379; 1:1000), HA (Roche; 12013819001; 1:500), 3 beta-hydroxysteroid dehydrogenase (HSD3B; Santa Cruz; sc-30820; 1:1000), and ACTB (Sigma; A3854; 1:100,000). The membranes were then probed with HRP-conjugated secondary antibodies (Jackson ImmunoResearch) and developed using Chemiluminescent HRP Substrate (Millipore).

### X-gal staining

As described [25], ovarian samples were first fixed in 2% paraformaldehyde-0.2% glutaraldehyde in a 0.1 M phosphate buffer solution (pH 7.4), and then stained with X-gal staining buffer containing 1 mg/ml X-gal, 5 mM potassium ferricyanide, and 5 mM potassium ferrocyanide. After the staining procedure, samples were post-fixed with 10% neutral buffered formalin for histological analysis. Fast red (Vector Laboratories) was used to counterstain the nucleus.

### RNAscope analysis

RNAscope 2.5 HD Reagent Kit and *Gli1* probe (Catalog no. 311001) were purchased from Advanced Cell Diagnostics. Positive control peptidylprolyl isomerase B (*Ppib*) and negative control dihydrodipicolinate reductase (*DapB*) probes were included. RNAscope in situ hybridization analysis was performed using formalin-fixed paraffin-embedded sections based on the manufacturer instructions. Hybridization signals were developed and visualized using DAB. Sections were counterstained using hematoxylin and mounted with Permount Mounting Medium.

### Statistical analyses

A two-tailed *t*-test (unpaired) was used to determine the difference of means between two groups. Data are mean ± standard error of the mean (s.e.m). Statistical significance was reported at ^∗^ *P* < 0.05, ^∗∗^ *P* < 0.01, or ^∗∗∗^ *P* < 0.001.

## Results

### Development of ovarian tumors in female mice harboring *TGFBR1*^*CA*^ conditional allele and *Gdf9*-iCre

**Fig. 1:**
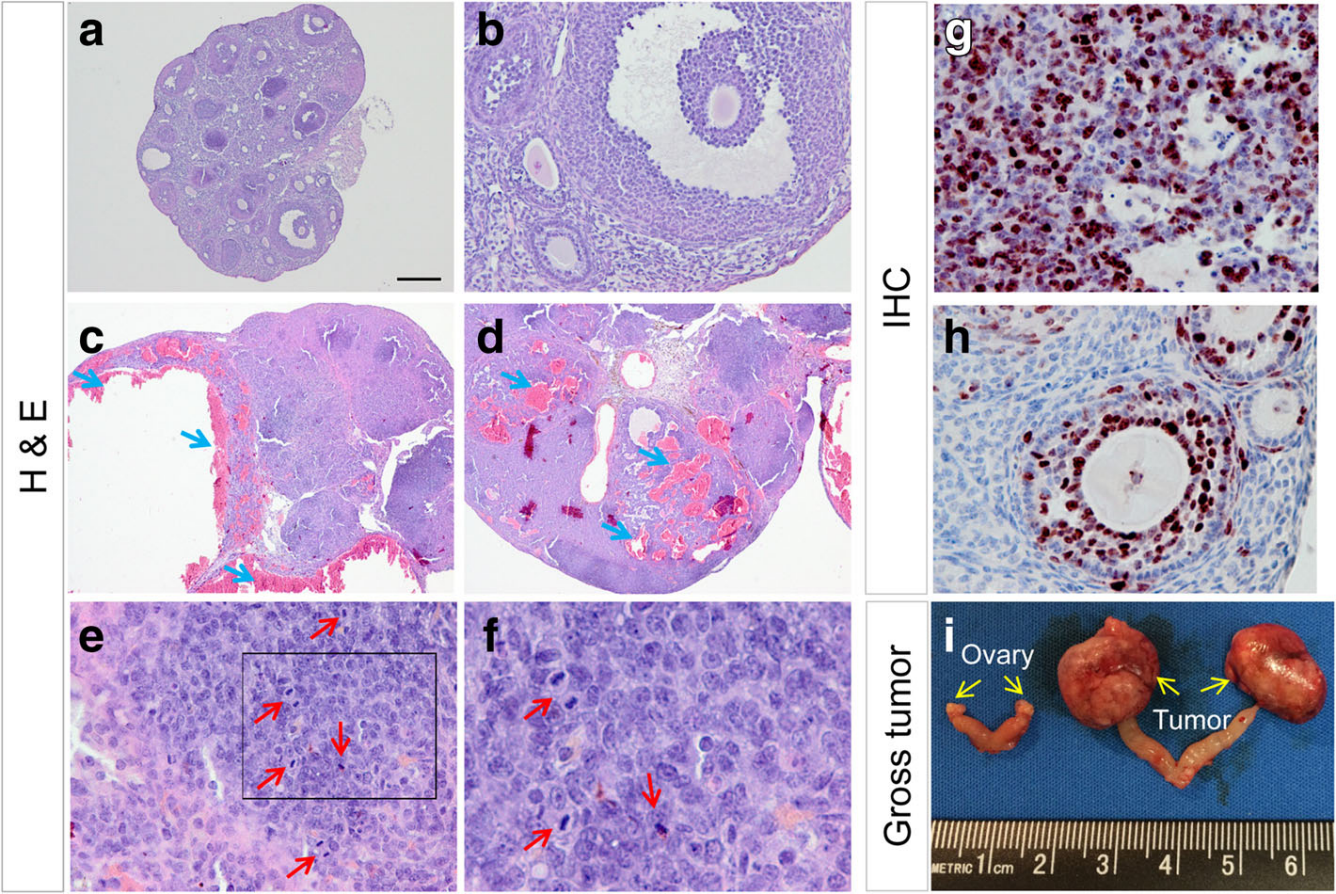
Development of sex cord-stromal tumors in TGFBR1-CA^G9Cre^ mice. **a-f** Histological analysis of 8-week-old control and TGFBR1-CA^G9Cre^ mice. Note the presence of hemorrhagic cysts (**c**; blue arrows) and hemorrhage (**d**; blue arrows) and neoplastic regions containing mitotic figures (**e** and **f**; red arrows) in TGFBR1-CA^G9Cre^ ovaries compared with control ovaries (**a** and **b**). Panel **f** is a higher magnification image for the boxed region in panel (**e**). **g** and **h** Immunohistochemical analysis of Ki67 using 8-week-old TGFBR1-CA^G9Cre^ (**g**) and control (**h**) ovaries. Experiment was performed using ABC method, and signals were developed using NovaRED Peroxidase Substrate Kit. Sections were counterstained with hematoxylin. Scale bar is representatively depicted in (**a**) and equals 12.5 μm (**f**), 25 μm (**e**, **g**, and **h**), 50 μm (**b**), and 250 μm (**a**, **c**, and **d**). H & E staining and immunohistochemistry were conducted using 3-5 independent samples per group. **i** Gross ovarian tumor morphology of a 7-month-old mouse. Yellow arrows denote the ovary and ovarian tumors in the control and TGFBR1-CA^G9Cre^ mice, respectively

The *TGFBR1* ^CA^ allele was engineered to contain three missense mutations to constitutively activate TGFBR1 and prevent the inhibitory effect of FK506 binding protein 1A (FKBP12) as previously described [29, 30]. To determine the potential phenotype of TGFBR1-CA^G9Cre^ mice, we first examined their fertility. During a 3-month fertility test, we found that TGFBR1-CA^G9Cre^ females (*n* = 3) were sterile, in contrast to wild type mice (8.25 ± 0.26 pups/litter and 1.22 ± 0.07 litter/month; *n* = 6). To define the cause of infertility, we examined the morphology of the ovary. Remarkably, we found the development of gross ovarian tumors in TGFBR1-CA^G9Cre^ mice at the age of 2-3 months. A summary of tumor development in mice at the age of 8-10 weeks is listed in Table 1. Histological analysis using H & E staining suggested the development of hemorrhagic GCTs in TGFBR1-CA^G9Cre^ mice compared with controls (Fig. 1a-d). The neoplastic cells appeared mitotically active (Fig. 1e and f). This observation was confirmed by high expression of Ki67, a cell proliferation marker, compared with controls (Fig. 1g and h). Figure 1i shows ovarian tumors in TGFBR1-CA^G9Cre^ mice at the age of 7 months.

**Table 1:** Development of ovarian tumors in TGFBR1-CA^G9Cre^ mice

Group	Age (week)	Number of animals (n)	Visible tumors (n)
Control	8-10	14	0
TGFBR1-CA^G9Cre^	8-10	15	15

### Perturbed follicular development at an early stage

**Fig. 2:**
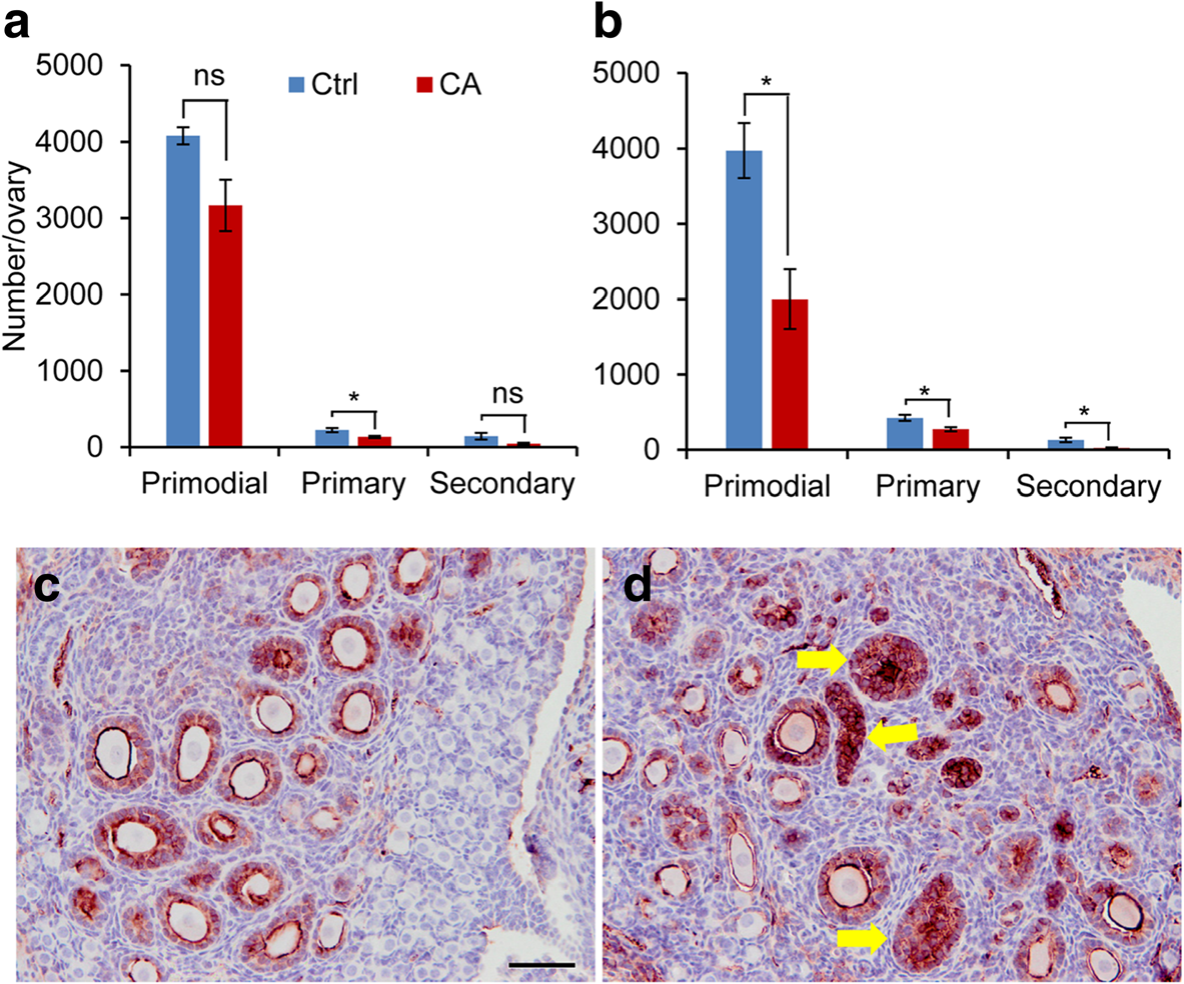
Altered follicular development in TGFBR1-CA^G9Cre^ mice. **a** and **b** Follicle counts of control and TGFBR1-CA^G9Cre^ ovaries at PD5 (**a**) and PD7 (**b**). Data are mean ± s.e.m. *n* = 3. ^*^ *P* < 0.05. Ns, not significant. **c** and **d** Immunohistochemical localization of INHA in PD7 control and TGFBR1-CA^G9Cre^ ovaries. Arrows indicate abnormal follicle structures. Experiment was performed using ABC method, and signals were developed using NovaRED Peroxidase Substrate Kit. Sections were counterstained with hematoxylin. Four independent samples per group were used for immunohistochemical analyses. Scale bar is representatively depicted in (**c**) and equals 50 μm (**c** and **d**)

**Fig. 3:**
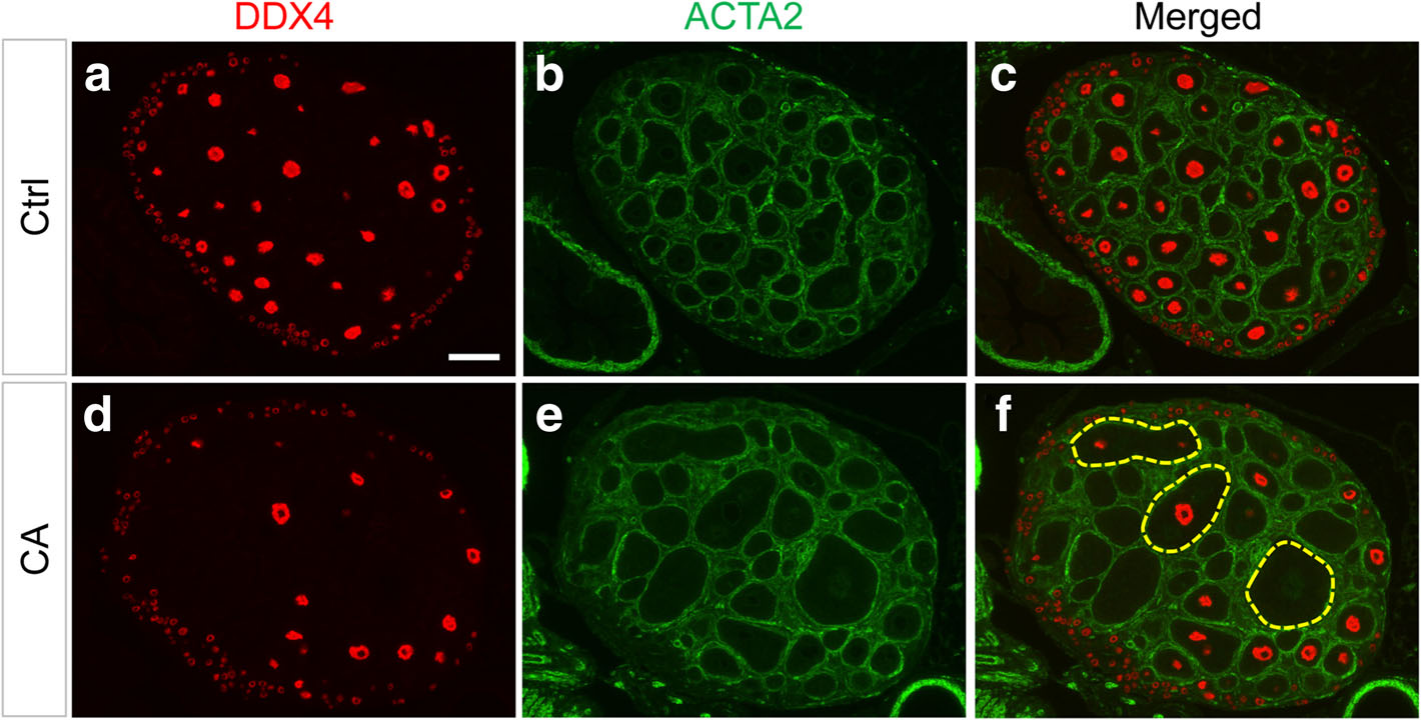
Immunofluorescence analysis of follicular defects in TGFBR1-CA^G9Cre^ mice. **a-f** Double immunofluorescence of DDX4 (red) and ACTA2 (green) using PD12 control (**a**-**c**) and TGFBR1-CA^G9Cre^ (**d**-**f**) mice. Three independent samples per group were analyzed using immunohistochemistry and/or immunofluorescence. Note the presence of large follicles or abnormal follicle-like structures (dotted yellow lines) in the ovaries of TGFBR1-CA^G9Cre^ mice in comparison with age-matched controls. Scale bar is representatively depicted in (**a**), and equals 100 μm (**a**-**f**)

To study the potential effect of TGFBR1 activation on follicular development, we performed follicle counting using ovaries from control and TGFBR1-CA^G9Cre^ mice at PD5 and PD7. The results did not reveal a significant difference in the number of primordial follicles between control and TGFBR1-CA^G9Cre^ mice at PD5, although a reduction of primary follicles was detected in the TGFBR1-CA^G9Cre^ mice (Fig. 2a). At PD7, the number of primordial follicles, primary follicles, and secondary follicles was reduced in TGFBR1-CA^G9Cre^ ovaries compared with controls (Fig. 2b). Meanwhile, large follicles or histologically abnormal follicle-like structures were found in the ovaries of TGFBR1-CA^G9Cre^ mice (Fig. 2c and d). Further double immunofluorescence staining of ACTA2 (a theca marker) and DDX4 (an oocyte marker) using PD12 ovaries independently confirmed these findings (Fig. 3a-f; dotted yellow lines). Ovarian histology of control and TGFBR1-CA^G9Cre^ mice at the age of PD7 and PD21 is shown in Additional file 1: Figure S1. These results suggest that constitutive activation of TGFBR1 using *Gdf9*-iCre disrupts ovarian follicle development, and the reduced follicle numbers in TGFBR1-CA^G9Cre^ mice may be associated with destruction/loss of follicles resulting from tumor initiation/development.

### Molecular analysis of ovarian tumor type

**Fig. 4:**
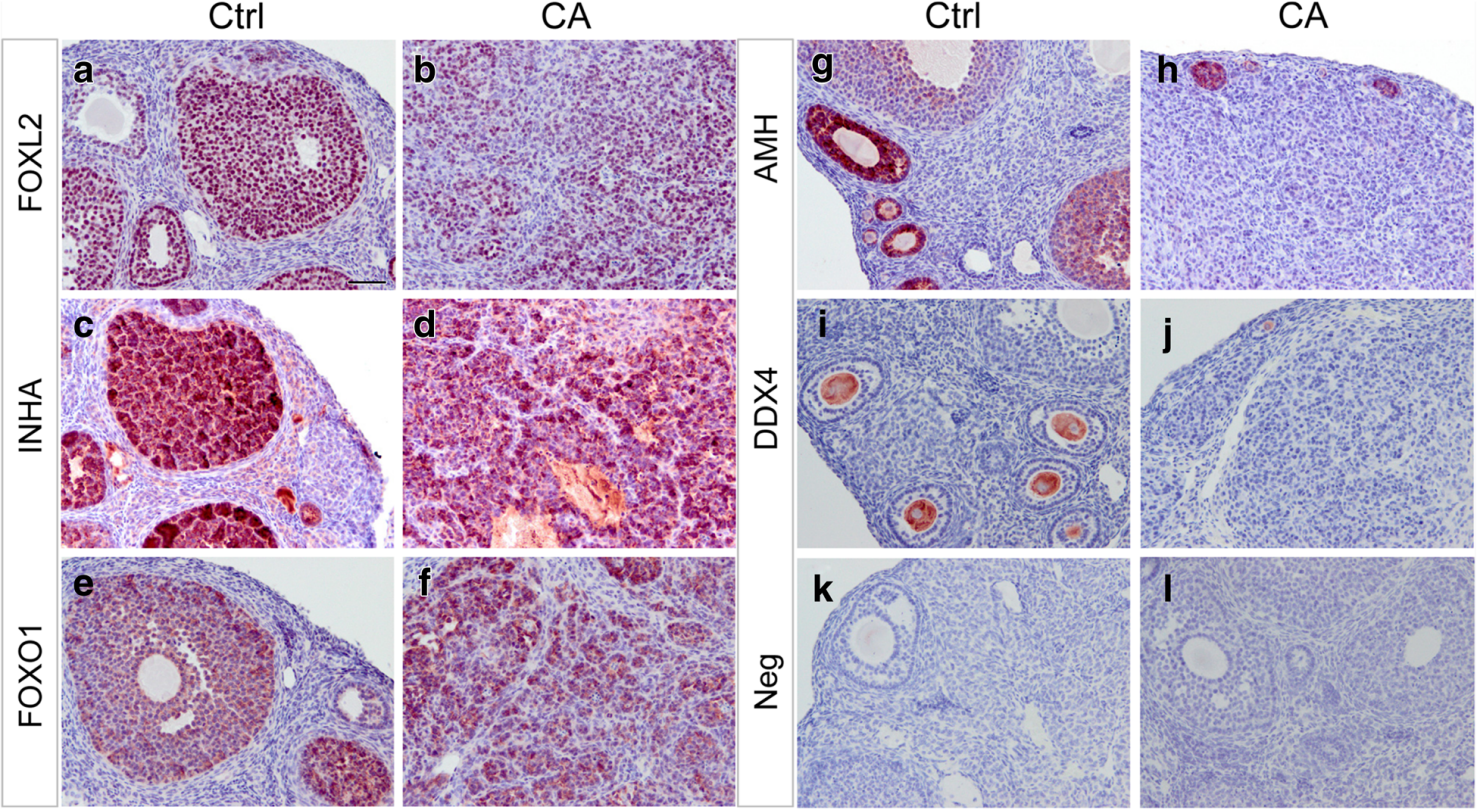
Immunohistochemical analysis of ovarian tumor markers. **a-j** Expression of granulosa cell and germ cell markers in 8-week-old control and TGFBR1-CA^G9Cre^ ovaries. Representative images from immunohistochemical analysis of FOXL2 (**a** and **b**), INHA (**c** and **d**), FOXO1 (**e** and **f**), AMH (**g** and **h**), and DDX4 (**i** and **j**) are shown. **k** and **l** Negative controls using isotype-matched rabbit and goat IgGs. Experiment was performed using ABC method, and signals were developed using NovaRED Peroxidase Substrate Kit. Sections were counterstained with hematoxylin. Five independent samples per group were utilized in this analysis. Scale bar is representatively depicted in (**a**) and equals 50 μm (**a**-**l**)

**Fig. 5:**
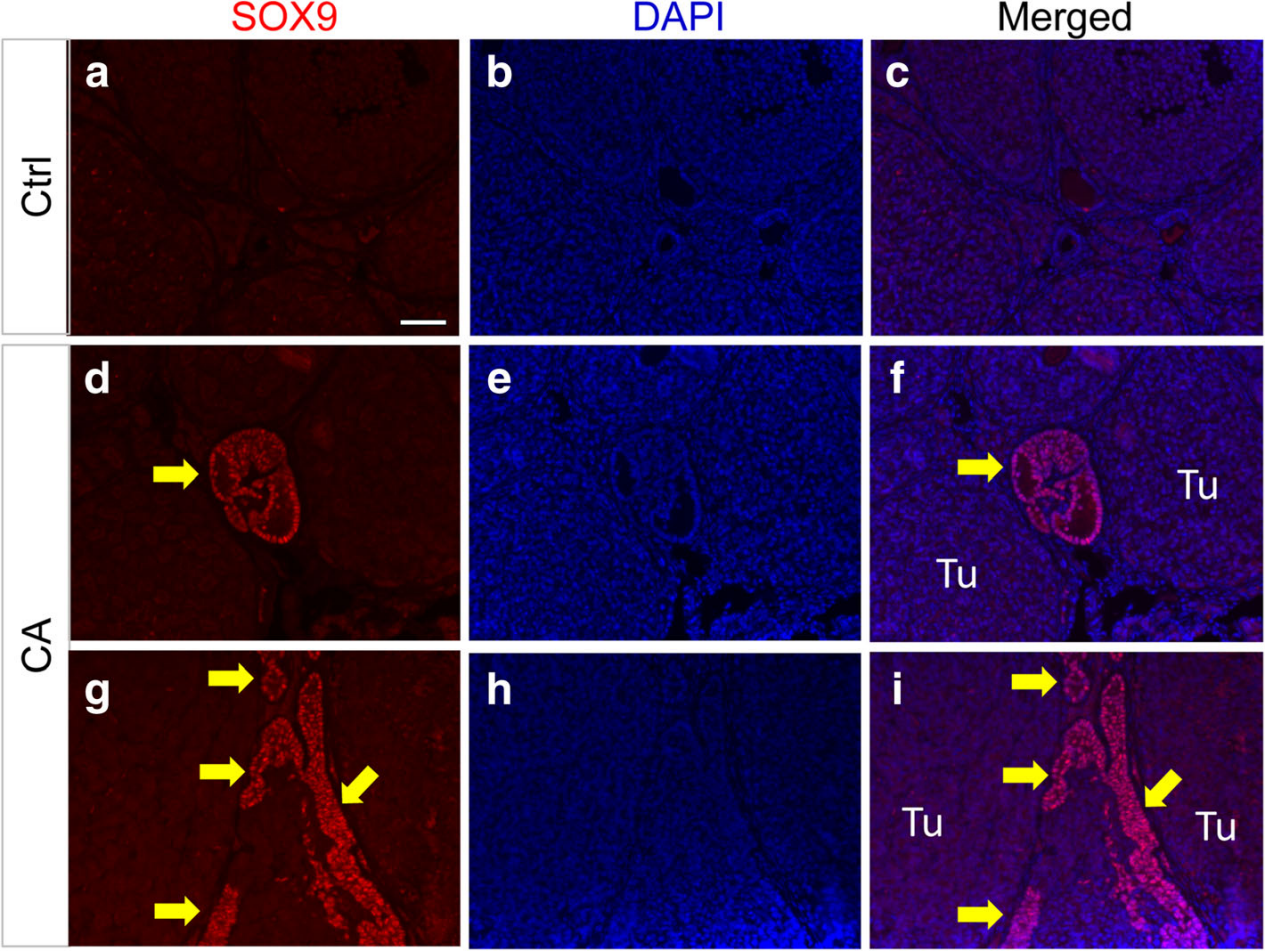
Immunofluorescence of SOX9 in control and TGFBR1-CA^G9Cre^ ovaries. **a-c** SOX9 expression in control ovaries. **d-i** SOX9 expression in TGFBR1-CA^G9Cre^ ovaries. DAPI (blue) was used to counterstain the nucleus. Arrows indicate abnormal expression of SOX9 proteins (red) in ovarian tumors. At least three control and TGFBR1-CA^G9Cre^ mice at the age of ~2 months were analyzed. The results of immunofluorescence were confirmed by immunohistochemistry (not shown). Tu, tumor. Scale bar is depicted in (**a**) and equals 50 μm (**a**-**i**)

To define the molecular characteristics of ovarian tumors in TGFBR1-CA^G9Cre^ mice, we performed immunostaining to examine the expression of a granulosa cell lineage marker FOXL2 [31] and three other granulosa cell-expressed proteins, FOXO1, INHA, and AMH. DDX4, a germ cell marker, was also included. The localization of FOXL2 (Fig. 4a), INHA (Fig. 4c), FOXO1 (Fig. 4e), AMH (Fig. 4g), and DDX4 (Fig. 4i) was detected in the granulosa cell or oocyte compartment of control ovaries, while ovarian tumor tissues from TGFBR1-CA^G9Cre^ mice were immunoreactive with FOXL2 (Fig. 4b), INHA (Fig. 4d), and FOXO1 (Fig. 4f), supporting the development of GCTs in these mice. However, immunoreactive signals of AMH were low to undetectable in these tumors (Fig. 4h), consistent with the reported lack of AMH expression in GCTs resulting from conditional deletion of FOXO1/3 and PTEN [32]. The tumor nodules did not express DDX4 (Fig. 4j). Representative negative controls using rabbit and goat IgGs are respectively shown in Fig. 4k and l. Because GCTs can express Sertoli cell genes [32], we examined whether SOX9 was detectable in TGFBR1-CA^G9Cre^ tumors. Our results showed heterogeneous expression of SOX9 in tumors from TGFBR1-CA^G9Cre^ mice (Fig. 5d-i; arrows) versus controls (Fig. 5a-c). Taken together, these findings support the histological observation that sustained activation of TGFBR1 using *Gdf9*-iCre leads to the formation of sex cord-stromal tumors reminiscent of GCTs.

### TGFBR1-CA^G9Cre^ mice did not show increased apoptosis in the oocyte

**Fig. 6:**
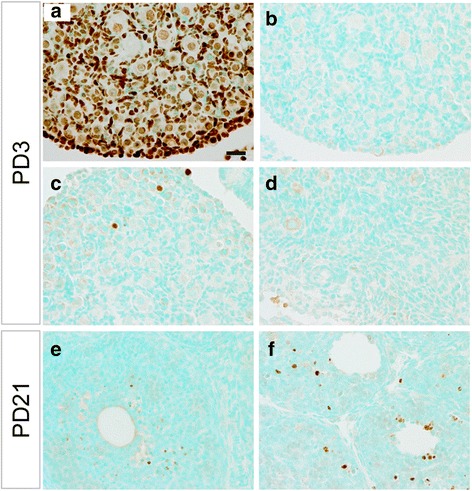
Apoptosis analysis of ovaries from control and TGFBR1-CA^G9Cre^ mice. **a** PD3 ovarian sections treated with DNase I as positive control. **b** Negative control where TdT was replaced with water. **c-f** Representative images of apoptosis analysis using PD3 (*n* = 4) and PD21 (*n* = 5) ovarian sections. Apoptotic cells were labeled with TdT and signals developed using DAB. Sections were counterstained with Methyl Green. Scale bar is representatively depicted in (**a**) and equals 20 μm (**a**-**f**)

It has been suggested that loss of oocytes during follicular development may alter the differentiation and cell fate of ovarian granulosa cells [33]. To determine whether TGFBR1 activation promotes oocyte apoptosis, we examined fragmentation/damage on DNA suggestive of apoptosis using an In situ Apoptosis Detection Kit and ovarian samples collected at PD3-PD21. To validate the experimental procedure, we showed that ovarian samples of positive controls treated with DNase I to generate fragmented DNA contained strong immunoreactive signals (Fig. 6a), while negative controls showed only background staining (Fig. 6b). The analysis did not show alterations of oocyte apoptosis in TGFBR1-CA^G9Cre^ ovaries at PD3-PD12 (Fig. 6c and d and data for PD5, PD7, and PD12 not shown). At PD21, however, signals were observed in ovarian somatic cells within some follicles in TGFBR1-CA^G9Cre^ mice compared with controls (Fig. 6e and f). Thus, these results indicate that oocyte apoptosis is not a main contributing factor to the overall ovarian pathology observed in the TGFBR1-CA^G9Cre^ mouse model.

### Evidence of TGFBR1 activation in ovarian granulosa cells of TGFBR1-CA^G9Cre^ mice

**Fig. 7:**
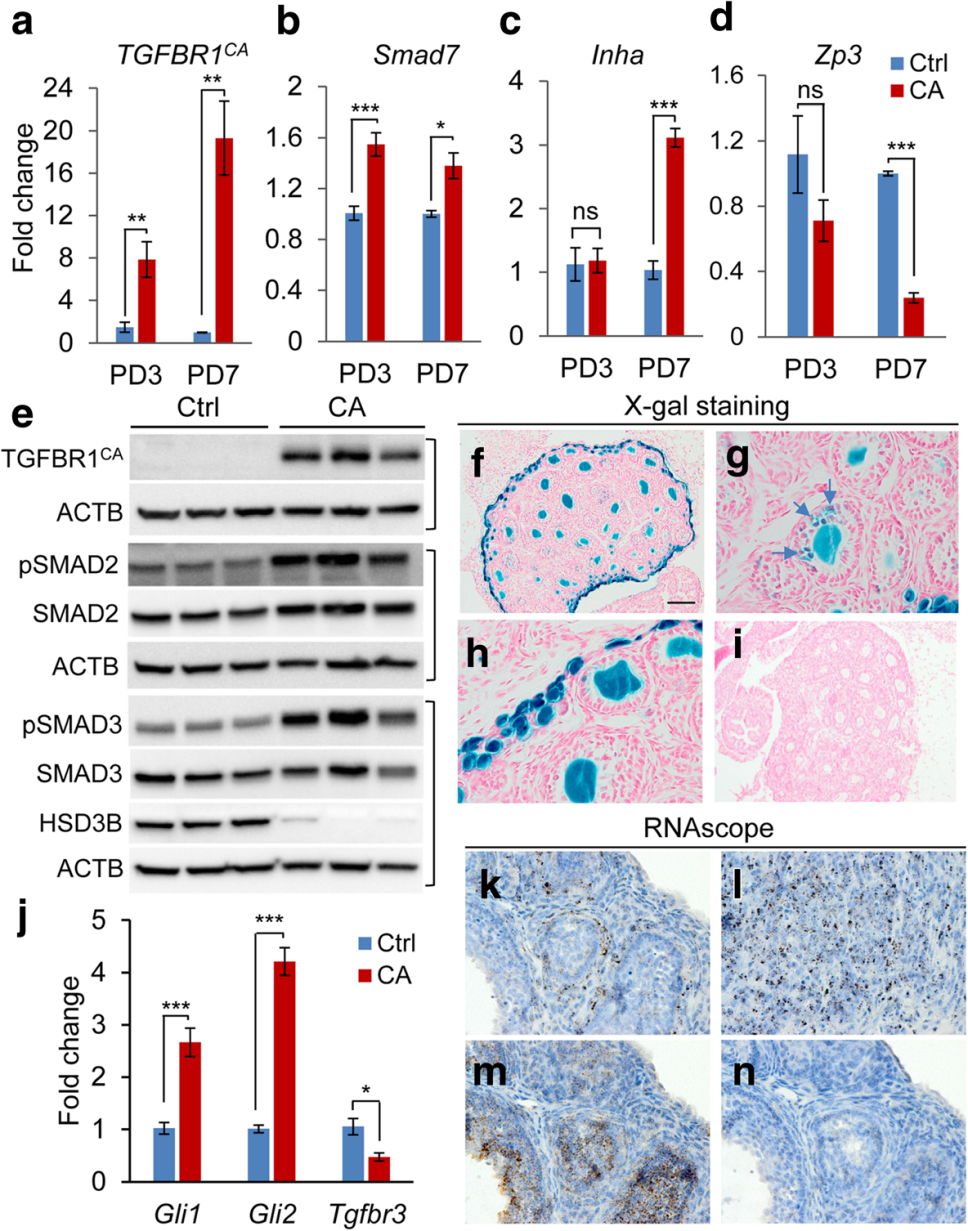
Evidence of TGFBR1 activation in ovarian granulosa cells of TGFBR1-CA^G9Cre^ mice. **a-d** Real-time PCR analysis of expression of *TGFBR1* ^CA^, *Smad7*, *Inha*, and *Zp3* in ovaries from control and TGFBR1-CA^G9Cre^ mice at PD3 and PD7. Real-time PCR was performed using ΔΔCT method. Data are mean ± s.e.m. *n* = 4-5. ^*^ *P* < 0.05, ^**^ *P* < 0.01, and ^***^ *P* < 0.001. Ns, not significant. **e** Western blotting analysis of TGFBR1^CA^, phospho-SMAD2/3, and HSD3B using ovaries from 2-month-old control and TGFBR1-CA^G9Cre^ mice. TGFBR1^CA^ was detected using an anti-HA antibody. *n* = 3. Each lane represents an independent sample. **f-i** X-gal staining using ovaries from *Rosa26*/*Gdf9*-iCre mice (**f**-**h**) and *Rosa26* control mice (**i**). Panels **g** and **h** are higher magnification images of two different fields of panel (**f**). At least 3 independent samples per group were used. **j** Real-time PCR analysis of the expression of *Gli1*, *Gli2*, and *Tgfbr3* using ovaries from 8-week-old control and TGFBR1-CA^G9Cre^ mice. Data are mean ± s.e.m. *n* = 4-5. ^*^ *P* < 0.05 and ^***^ *P* < 0.001. **k-n** RNAscope in situ hybridization analysis of *Gli1* mRNA distribution using 8-week-old control (**k**) and TGFBR1-CA^G9Cre^ ovaries (**l**). *n* = 4. Positive and negative controls using *TGFBR1* ^CA flox/+^ ovaries were shown in (**m**) and (**n**), respectively. Sections were counterstained with hematoxylin. Scale bar is representatively shown in (**f**) and equals 25 μm (**g**, **h**, and **k**-**n**) and 100 μm (**f** and **i**)

In line with the expression of *Gdf9*-iCre in primordial follicles from PD3 [17], *TGFBR1* ^CA^ transcripts were highly expressed in the ovaries of TGFBR1-CA^G9Cre^ mice (Fig. 7a), coinciding with increased expression of *Smad7*, a TGFB target gene (Fig. 7b). Moreover, the mRNA expression of *Inha* was increased and *Zp3* reduced at PD7 (Fig. 7c and d), corroborating altered granulosa cell and oocyte properties. Somewhat unexpected, our further analysis showed that ovarian tumor tissues which barely contained oocytes (Fig. 4j) readily expressed TGFBR1^CA^, along with increased levels of phospho-SMAD2/3 and reduced expression of HSD3B in comparison to controls (Fig. 7e). Activation of TGFB signaling was also examined using ovarian samples from 1-month-old mice (Additional file 2: Figure S2). These results suggest overactivation of TGFB signaling in GCT cells and alteration of cell differentiation.

The aforementioned findings raised the question on whether overactivation of TGFBR1 in granulosa cells conferred the development of ovarian tumors in TGFBR1-CA^G9Cre^ mice. To verify the expression of *Gdf9*-iCre in female germ cells, we performed X-gal staining using ovaries from *Rosa26*/*Gdf9*-iCre mice and demonstrated predominant Cre activity in oocytes from the primordial stage (Fig. 7f-h). However, minor sporadic signals were observed in some follicles (Fig. 7g; arrows), and the ovarian surface also appeared to be stained (Fig. 7h). As a quality control, ovaries from *Rosa26/Zp3*-Cre mice were included, where X-gal staining was exclusively observed in growing oocytes (Additional file 3: Figure S3 A and B). Negative controls using ovaries from *Rosa26* reporter mice alone did not show X-gal staining (Fig. 7i). Moreover, higher levels of *Gli1* and *Gli2* transcription factors and lower levels of *Tgfbr3* were found in TGFBR1-CA^G9Cre^ ovaries versus controls by real-time PCR (Fig. 7j). RNAscope in situ hybridization analysis further revealed the strong localization of *Gli1* mRNA to GCT tissues (Fig. 7l), in contrast to the restricted expression pattern of *Gli1* mRNA to theca layers in control ovaries (Fig. 7k). Representative positive and negative controls for the RNAscope experiment are respectively depicted in Fig. 7m and n.

## Discussion

GCTs are the major type of sex cord-stromal tumors, and both genomic and genetic factors are involved in the pathogenesis of these tumors [34, 35]. Human GCTs can be divided into adult and juvenile types; the former represents more than 90% of all GCTs and mainly occurs in perimenopausal or postmenopausal women around 50-54 years of age [36]. The Juvenile GCTs are relatively rare and mainly occur in prepubertal girls [37]. Although the 5-year survival rate in stage I patients is generally high, a poor prognosis occurs in patients with advanced stages of disease [38–40]. Of note, human adult GCTs have a high risk of recurrence [41], leading to resistance to chemotherapy and death. Because of the rarity of this category of tumors, mouse models are beneficial to study the etiopathology of these tumors.

Several reports using genetically modified mouse models highlight the importance of TGFB superfamily signaling components, such as INHA, SMAD3, SMAD1/5, and BMPR1A/BMPR1B, in sex cord-stromal tumor development [8–11, 42, 43]. A causal link between the mRNA expression levels of TGFBR1 and the development of GCTs in humans has not been established [43]. Growing evidence supports that activation of TGFB/activin signaling may represent a driving force of GCT development in mice [44]. It has also been found that TGFB/activin signaling is active in human GCTs [32, 45]. Notably, nearly all adult GCTs bear a somatic missense mutation of *FOXL2* C→G (C134W) [12], which may alter activin/TGFB and BMP signaling activity and the proliferation and differentiation status of granulosa cells [46–48]. In an effort to define the role of aberrant activation of TGFB signaling in the pathogenesis of ovarian tumors, we utilized a mouse model harboring constitutively active TGFBR1. Our results corroborate the role of TGFB signaling in ovarian tumorigenesis evidenced by the finding that constitutive activation of TGFB signaling in ovarian somatic cells leads to the formation of ovarian malignancies that phenocopy GCTs in several perspectives [16]. Of note, sustained activation of TGFBR1 led to the phosphorylation of SMAD2/3, which are downstream signaling elements shared by TGFBs and activins. Therefore, our results do not discount the role of activin signaling in GCT development. Indeed, our early studies have shown that activin signaling is important in promoting ovarian sex cord-stromal tumor development [11, 42]. Collectively, our approach to manipulate TGFBR1 activity and SMAD2/3 activation represents a valuable tool to study ovarian GCT development.

Oocyte-somatic cell communication is critical for follicular development. Through the secretion of growth factors, some of which are TGFB superfamily proteins, oocytes regulate folliculogenesis in a bi-directional paradigm [13, 14]. The involvement and potential role of oocyte-granulosa cell regulatory loop in sex cord-stromal tumor development are poorly understood. A recent study has provided functional evidence that constitutive activation of PI3K-AKT signaling in the oocyte promotes GCT formation [15], suggesting that dysregulation of major growth regulatory pathways in the oocyte impacts granulosa cell growth and differentiation. Genetic evidence suggests that SMAD4-dependent canonical TGFB signaling in oocytes is largely dispensable for female fertility [49]. However, it has not been determined whether unopposed TGFB signaling in the oocyte is detrimental to ovarian development and function. In the current study, ovarian tumors developed in mice harboring *TGFBR1* ^CA^ and *Gdf9*-iCre. By performing histological and molecular analyses, we demonstrated that the ovarian neoplasms were sex cord-stromal tumors which expressed granulosa cell markers including FOXL2, INHA, and FOXO1. An elegant review by Pitman and colleagues suggests that premature loss of ovarian germ cells may promote the formation of ovarian neoplasms [33]. However, the tumor phenotypes appear to be associated with the type of mutations and the onset of oocyte loss [33]. In this study, the specific cause of follicle/oocyte reduction and its potential involvement in ovarian tumor development remain unknown. The observation that overactivation of TGFBR1 using *Gdf9*-iCre did not increase oocyte apoptosis suggests that the reduction of follicle/oocyte numbers in TGFBR1-CA^G9Cre^ mice may be associated with the disruptive effect (e.g., impairment/destruction) of tumor formation and development.

The development of GCTs in TGFBR1-CA^G9Cre^ mice begs the question of how overactivation of TGFBR1 links to ovarian tumor formation. As a first step, expression of *Gdf9*-iCre was verified in our model. Our X-gal staining showed predominant Cre activity in the oocyte. Somewhat unexpectedly, we also observed minor sporadic signals in some follicles. Of note, in TGFBR1-CA^G9Cre^ mice, *Gdf9*-iCre was transmitted maternally due to the extremely low efficiency to generate *TGFBR1* ^CA flox/+^; *Gdf9*-iCre mice using male breeders (i.e., *Gdf9*-iCre males) to transmit *Gdf9*-iCre. In our breeding strategy, *Gdf9*-iCre females were crossed with *TGFBR1* ^CA flox/+^ male mice to produce *TGFBR1* ^CA flox/+^; *Gdf9*-iCre females. The reason for the inability of obtaining *TGFBR1* ^CA flox/+^; *Gdf9*-iCre mice using males to transmit *Gdf9*-iCre was not clear. Since constitutively active TGFBR1 in ovarian somatic cells is a strong driver of ovarian GCTs in mice [16], it was possible that even low to negligible Cre activity in a subset of granulosa cells might be permissive for *TGFBR1* ^CA^ activation, leading to GCT development. Further supporting the contribution of overactivation of TGFBR1 in granulosa cells to ovarian tumorigenesis in the TGFBR1-CA^G9Cre^ model, mice harboring *TGFBR1* ^CA^ and *Zp3*-Cre, which is expressed in growing oocytes, but not non-growing oocytes of primordial follicles [19], did not develop ovarian tumors, regardless of the parental origin of Cre transmission (Y. Gao and Q. Li, unpublished observation). We previously found that ovarian GCTs induced by overactivation of TGFBR1 in granulosa cells express higher levels of *Gli1* and *Gli2* transcription factors and lower levels of *Tgfbr3* compared with normal ovaries [16]. Interestingly, a similar transcript expression pattern of these genes was found in the ovaries of TGFBR1-CA^G9Cre^ mice. These findings collectively suggest that ovarian tumor formation in TGFBR1-CA^G9Cre^ mice may be attributable, at least partially, to overactivation of TGFBR1 in the granulosa cell compartment. Since the *TGFBR1* ^CA^ is tagged with HA, we have tested the utility of a number of commercially available antibodies directed to HA in immunohistochemical/immunofluorescence applications, with the aim of identifying malignant granulosa cells harboring TGFBR1 overactivation. Although some antibodies performed reasonably in western blotting analysis, none of them generated reproducible and convincing results in immunohistochemical/immunofluorescence assays. Future work using *Rosa26*/*TGFBR1* ^CA^/*Gdf9*-iCre mice may help elucidate the cellular origin of TGFBR1 overactivation and its contribution to ovarian GCT formation. It is worthwhile mentioning that a potential contribution of overactivation of TGFBR1 in the primordial oocyte or in both primordial oocytes and granulosa cells to GCT development could not be excluded. Further clarification of this question relays on the technical capability of specifically manipulating TGFB signaling in the oocyte of primordial follicles.

In summary, this study has created a mouse model of GCTs with defined disease onset that can be further exploited to study the role of TGFB signaling in ovarian tumor development. Of note, our studies were performed using mice and extrapolation of the findings to human GCTs needs further investigation. Identification of molecular markers for GCTs would benefit early diagnosis and treatment. To date, there are no reliable biomarkers for GCTs, although serum levels of estradiol, inhibin, and AMH have been extensively investigated and appear to correlate with GCTs in some cases [50]. Ongoing studies are to identify molecular signatures of GCTs in our model during tumor initiation and development.

## Conclusions

Results of the current study reinforce the role of constitutively active TGFBR1 in promoting ovarian tumorigenesis in mice. The mouse model created in this study may be further exploited to define the cellular and molecular mechanisms of TGFB/activin downstream signaling in GCT development. Future studies are needed to test whether activation of TGFB/activin signaling contributes to the development of human GCTs.

## Additional files

Additional file 1: Figure S1. Histological and morphological analysis of ovaries from immature TGFBR1-CA^G9Cre^ and control mice. (**A**-**H**) Periodic acid Schiff’s staining of ovarian samples from TGFBR1-CA^G9Cre^ and control mice at PD7 and PD21. Panels (B, D, F, and H) represent higher magnification images for the corresponding panels (A, C, E, and G). Scale bar is representatively shown in (A) and equals 25 μm (B, D, F, and H) and 100 μm (A, C, E, and G). (I) Reproductive tract of TGFBR1-CA^G9Cre^ and control mice at PD21. OV, ovary; Ut, uterus. Scale bar = 10 mm (TIFF 3522 kb)

Additional file 2: Figure S2. Western blotting analysis of phospho-SMAD2/3 and TGFBR1^CA^ using ovaries from 1-month-old control and TGFBR1-CA^G9Cre^ mice. Note that a pronounced increase in phospho-SMAD2 but not phospho-SMAD3 was observed at this stage, suggesting that SMAD3 activation may have a later onset or is masked by high levels of phospho-SMAD3 within control ovaries at this stage. TGFBR1^CA^ was detected using an anti-HA antibody. ACTB was included as internal control. *n* = 3-4. Each lane represents an independent sample (TIFF 366 kb)

Additional file 3: Figure S3. Reporter analysis of *Zp3*-Cre activity in the ovary. (**A** and **B**) X-gal staining of ovaries from *Rosa26*/*Zp3*-Cre mice at PD14. Panel (B) is a higher magnification image of panel (A). Results represent staining using 3 independent samples. Scale bar is representatively shown in (A) and equals 25 μm (B) and 100 μm (A) (TIFF 1070 kb)

## Abbreviations

ABC: Avidin-biotin complex
ACTA2: Smooth muscle actin alpha
AMH: Anti-Mullerian hormone
BMP: Bone morphogenetic protein
DAB: 3,3′-diaminobenzidine
DAPB: Dihydrodipicolinate reductase
DAPI: 4′,6-diamidino-2-phenylindole
DDX4: DEAD (Asp-Glu-Ala-Asp) box polypeptide 4
FKBP12: FK506 binding protein 1A
FOXL2: Forkhead box L2
FOXO1: Forkhead box O1
GCTs: Granulosa cell tumors
GDF9: Growth differentiation factor 9
GLI1: GLI-Kruppel family member GLI1
H & E: Hematoxylin and eosin
HPRT: Hypoxanthine guanine phosphoribosyl transferase
HRP: Horseradish peroxidase
HSD3B: 3 beta-hydroxysteroid dehydrogenase
IgGs: Immunoglobulin Gs
INHA: Inhibin alpha
PAS: Periodic acid Schiff’s
PD: Postnatal day
PI3K: Phosphoinositide-3-kinase
PPIB: Peptidylprolyl isomerase B
RPL19: Ribosomal protein L19
SOX9: SRY (sex determining region Y)-box 9
TdT: Terminal deoxynucleotidyl transferase
TGFB: Transforming growth factor beta
TGFBR1^CA^: Constitutively active TGFBR1
TGFBR1-CA^G9Cre^: *TGFBR1* ^CA flox/+^; *Gdf9*-iCre
ZP3: Zona pellucida glycoprotein 3

## References

[1] Massague J (2012). TGFbeta signalling in context. Nat Rev Mol Cell Biol.

[2] Knight PG, Glister C (2006). TGF-beta superfamily members and ovarian follicle development. Reproduction.

[3] Li Q (2014). Transforming growth factor beta signaling in uterine development and function. J Anim Sci Biotechnol.

[4] Laiho M, DeCaprio JA, Ludlow JW, Livingston DM, Massague J (1990). Growth inhibition by TGF-beta linked to suppression of retinoblastoma protein phosphorylation. Cell.

[5] Principe DR, Doll JA, Bauer J, Jung B, Munshi HG, Bartholin L, Pasche B, Lee C, Grippo PJ (2014). TGF-beta: duality of function between tumor prevention and carcinogenesis. J Natl Cancer Inst.

[6] Welch DR, Fabra A, Nakajima M (1990). Transforming growth-factor-beta stimulates mammary adenocarcinoma cell invasion and metastatic potential. Proc Natl Acad Sci U S A.

[7] Chuvin N, Vincent DF, Pommier RM, Alcaraz LB, Gout J, Caligaris C, Yacoub K, Cardot V, Roger E, Kaniewski B (2017). Acinar-to-ductal metaplasia induced by transforming growth factor beta facilitates KRASG12D-driven pancreatic tumorigenesis. Cell Mol Gastroenterol Hepatol.

[8] Matzuk MM, Finegold MJ, Su JG, Hsueh AJ, Bradley A (1992). Alpha-inhibin is a tumour-suppressor gene with gonadal specificity in mice. Nature.

[9] Pangas SA, Li X, Umans L, Zwijsen A, Huylebroeck D, Gutierrez C, Wang D, Martin JF, Jamin SP, Behringer RR (2008). Conditional deletion of Smad1 and Smad5 in somatic cells of male and female gonads leads to metastatic tumor development in mice. Mol Cell Biol.

[10] Edson MA, Nalam RL, Clementi C, Franco HL, Demayo FJ, Lyons KM, Pangas SA, Matzuk MM (2010). Granulosa cell-expressed BMPR1A and BMPR1B have unique functions in regulating fertility but act redundantly to suppress ovarian tumor development. Mol Endocrinol.

[11] Li Q, Graff JM, O'Connor AE, Loveland KL, Matzuk MM (2007). SMAD3 regulates gonadal tumorigenesis. Mol Endocrinol.

[12] Shah SP, Kobel M, Senz J, Morin RD, Clarke BA, Wiegand KC, Leung G, Zayed A, Mehl E, Kalloger SE (2009). Mutation of FOXL2 in granulosa-cell tumors of the ovary. N Engl J Med.

[13] Eppig JJ, Chesnel F, Hirao Y, O'Brien MJ, Pendola FL, Watanabe S, Wigglesworth K (1997). Oocyte control of granulosa cell development: how and why. Hum Reprod.

[14] Eppig JJ (2001). Oocyte control of ovarian follicular development and function in mammals. Reproduction.

[15] Kim SY, Ebbert K, Cordeiro MH, Romero MM, Whelan KA, Suarez AA, Woodruff T, Kurita T (2016). Constitutive activation of PI3K in oocytes induces ovarian granulosa cell tumors. Cancer Res.

[16] Gao Y, Vincent DF, Davis AJ, Sansom OJ, Bartholin L, Li Q (2016). Constitutively active transforming growth factor beta receptor 1 in the mouse ovary promotes tumorigenesis. Oncotarget.

[17] Lan ZJ, Xu X, Cooney AJ (2004). Differential oocyte-specific expression of Cre recombinase activity in GDF-9-iCre, Zp3cre, and Msx2Cre transgenic mice. Biol Reprod.

[18] Andreu-Vieyra CV, Chen RH, Agno JE, Glaser S, Anastassiadis K, Stewart AF, Matzuk MM (2010). MLL2 is required in oocytes for bulk histone 3 lysine 4 trimethylation and transcriptional silencing. PLoS Biol.

[19] de Vries WN, Binns LT, Fancher KS, Dean J, Moore R, Kemler R, Knowles BB (2000). Expression of Cre recombinase in mouse oocytes: a means to study maternal effect genes. Genesis.

[20] Soriano P (1999). Generalized lacZ expression with the ROSA26 Cre reporter strain. Nat Genet.

[21] Bartholin L, Cyprian FS, Vincent D, Garcia CN, Martel S, Horvat B, Berthet C, Goddard-Leon S, Treilleux I, Rimokh R, Marie JC (2008). Generation of mice with conditionally activated transforming growth factor Beta signaling through the T beta RI/ALK5 receptor. Genesis.

[22] Vincent DF, Kaniewski B, Powers SE, Havenar-Daughton C, Marie JC, Wotton D, Bartholin L (2010). A rapid strategy to detect the recombined allele in LSL-TbetaRICA transgenic mice. Genesis.

[23] Bristol-Gould SK, Kreeger PK, Selkirk CG, Kilen SM, Cook RW, Kipp JL, Shea LD, Mayo KE, Woodruff TK (2006). Postnatal regulation of germ cells by activin: the establishment of the initial follicle pool. Dev Biol.

[24] Myers M, Britt KL, Wreford NG, Ebling FJ, Kerr JB (2004). Methods for quantifying follicular numbers within the mouse ovary. Reproduction.

[25] Li Q, Agno JE, Edson MA, Nagaraja AK, Nagashima T, Matzuk MM (2011). Transforming growth factor beta receptor type 1 is essential for female reproductive tract integrity and function. PLoS Genet.

[26] Gao Y, Wen H, Wang C, Li Q (2013). SMAD7 antagonizes key TGFbeta superfamily signaling in mouse granulosa cells in vitro. Reproduction.

[27] Spandidos A, Wang XW, Wang HJ, Seed B (2010). PrimerBank: a resource of human and mouse PCR primer pairs for gene expression detection and quantification. Nucleic Acids Res.

[28] Livak KJ, Schmittgen TD (2001). Analysis of relative gene expression data using real-time quantitative PCR and the 2(−Delta Delta C(T)) method. Methods.

[29] Wieser R, Wrana JL, Massague J (1995). Gs domain mutations that constitutively activate T-Beta-R-I, the downstream signaling component in the Tgf-Beta receptor complex. EMBO J.

[30] Charng MJ, Kinnunen P, Hawker J, Brand T, Schneider MD (1996). FKBP-12 recognition is dispensable for signal generation by type I transforming growth factor-beta receptors. J Biol Chem.

[31] Schmidt D, Ovitt CE, Anlag K, Fehsenfeld S, Gredsted L, Treier AC, Treier M (2004). The murine winged-helix transcription factor Foxl2 is required for granulosa cell differentiation and ovary maintenance. Development.

[32] Liu Z, Ren YA, Pangas SA, Adams J, Zhou W, Castrillon DH, Wilhelm D, Richards JS (2015). FOXO1/3 and PTEN depletion in granulosa cells promotes ovarian granulosa cell tumor development. Mol Endocrinol.

[33] Pitman JL, Mcneilly AS, Mcneilly JR, Hays LE, Bagby GC, Sawyer HR, Mcnatty KP (2012). The fate of granulosa cells following premature oocyte loss and the development of ovarian cancers. Int J Dev Biol.

[34] Jamieson S, Fuller PJ (2012). Molecular pathogenesis of granulosa cell tumors of the ovary. Endocr Rev.

[35] Fuller PJ, Leung D, Chu S (2017). Genetics and genomics of ovarian sex cord-stromal tumors. Clin Genet.

[36] Schumer ST, Cannistra SA (2003). Granulosa cell tumor of the ovary. J Clin Oncol.

[37] Young RH, Dickersin GR, Scully RE (1984). Juvenile granulosa cell tumor of the ovary. A clinicopathological analysis of 125 cases. Am J Surg Pathol.

[38] Evans AT, Gaffey TA, Malkasian GD, Annegers JF (1980). Clinicopathologic review of 118 granulosa and 82 theca cell tumors. Obstet Gynecol.

[39] Bjorkholm E, Silfversward C (1981). Prognostic factors in granulosa-cell tumors. Gynecol Oncol.

[40] Malmstrom H, Hogberg T, Risberg B, Simonsen E (1994). Granulosa cell tumors of the ovary: prognostic factors and outcome. Obstet Gynecol.

[41] Miller K, McCluggage WG (2008). Prognostic factors in ovarian adult granulosa cell tumour. J Clin Pathol.

[42] Li Q, Kumar R, Underwood K, O'Connor AE, Loveland KL, Seehra JS, Matzuk MM (2007). Prevention of cachexia-like syndrome development and reduction of tumor progression in inhibin-deficient mice following administration of a chimeric activin receptor type II-murine Fc protein. Mol Hum Reprod.

[43] Mansouri-Attia N, Tripurani SK, Gokul N, Piard H, Anderson ML, Eldin K, Pangas SA (2014). TGFbeta signaling promotes juvenile granulosa cell tumorigenesis by suppressing apoptosis. Mol Endocrinol.

[44] Fang X, Gao Y, Li Q (2016). SMAD3 activation: a converging point of dysregulated TGF-Beta superfamily signaling and genetic aberrations in granulosa cell tumor development?. Biol Reprod.

[45] Middlebrook BS, Eldin K, Li X, Shivasankaran S, Pangas SA (2009). Smad1-Smad5 ovarian conditional knockout mice develop a disease profile similar to the juvenile form of human granulosa cell tumors. Endocrinology.

[46] Nonis D, McTavish KJ, Shimasaki S (2013). Essential but differential role of FOXL2(wt) and FOXL2(C134W) in GDF-9 stimulation of follistatin transcription in co-operation with Smad3 in the human granulosa cell line COV434. Mol Cell Endocrinol.

[47] Cheng JC, Chang HM, Qiu X, Fang L, Leung PC (2014). FOXL2-induced follistatin attenuates activin A-stimulated cell proliferation in human granulosa cell tumors. Biochem Biophys Res Commun.

[48] Rosario R, Araki H, Print CG, Shelling AN (2012). The transcriptional targets of mutant FOXL2 in granulosa cell tumours. PLoS One.

[49] Li X, Tripurani SK, James R, Pangas SA (2012). Minimal fertility defects in mice deficient in oocyte-expressed Smad4. Biol Reprod.

[50] Colombo N, Parma G, Zanagnolo V, Insinga A (2007). Management of ovarian stromal cell tumors. J Clin Oncol.

