# Supplementary figures and images for: Disruption of postnatal folliculogenesis and development of ovarian tumor in a mouse model with aberrant transforming growth factor beta signaling
Source: Reprod Biol Endocrinol. 2017 Dec 8;15:94. doi: 10.1186/s12958-017-0312-z (PMC5723096; doi:10.1186/s12958-017-0312-z)

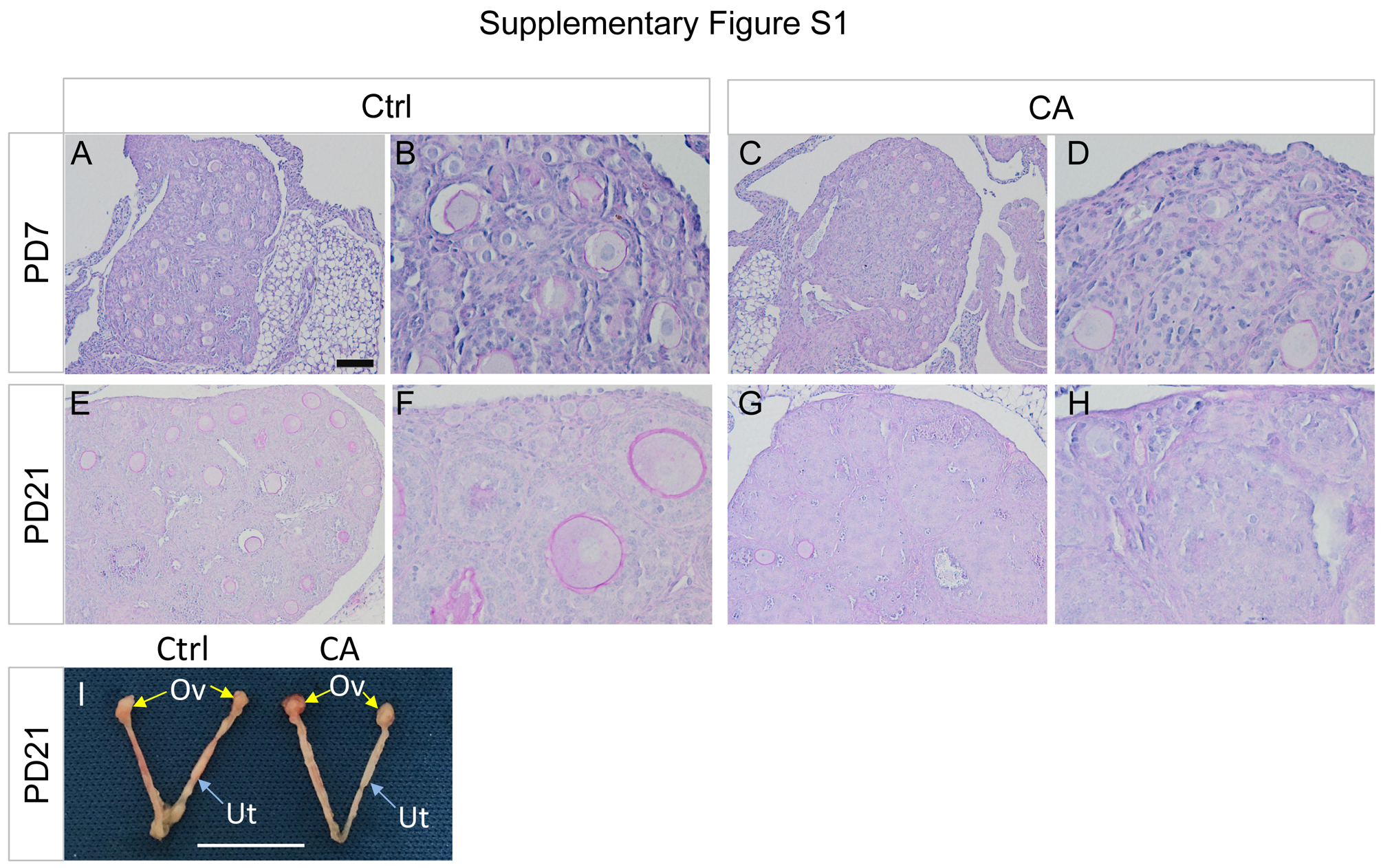

Supplement: Supplementary file 1 — Histological and morphological analysis of ovaries from immature TGFBR1-CAG9Cre and control mice. (A-H) Periodic acid Schiff’s staining of ovarian samples from TGFBR1-CAG9Cre and control mice at PD7 and PD21. Panels (B, D, F, and H) represent higher magnification images for the corresponding panels (A, C, E, and G). Scale bar is representatively shown in (A) and equals 25 μm (B, D, F, and H) and 100 μm (A, C, E, and G). (I) Reproductive tract of TGFBR1-CAG9Cre and control mice at PD21. OV, ovary; Ut, uterus. Scale bar = 10 mm (TIFF 3522 kb) [file 12958_2017_312_MOESM1_ESM.tif]

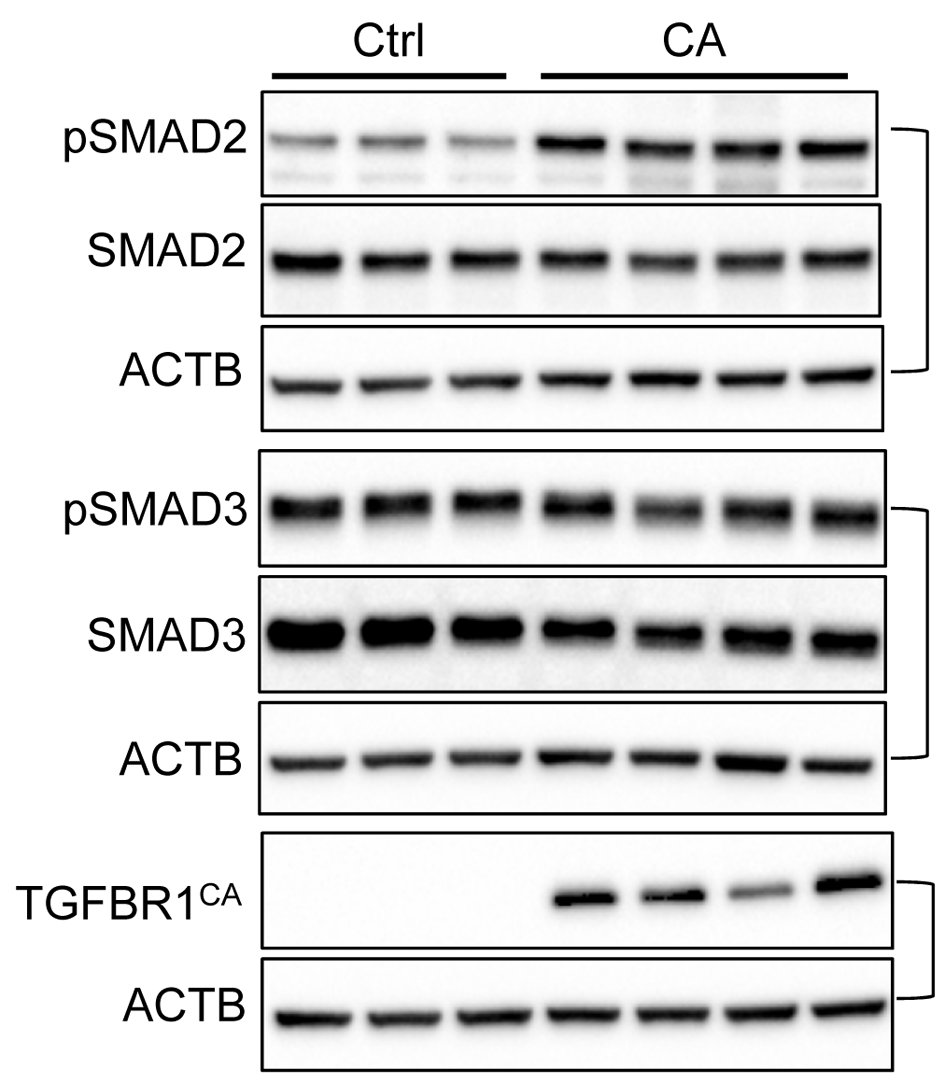

Supplement: Supplementary file 2 — Western blotting analysis of phospho-SMAD2/3 and TGFBR1CA using ovaries from 1-month-old control and TGFBR1-CAG9Cre mice. Note that a pronounced increase in phospho-SMAD2 but not phospho-SMAD3 was observed at this stage, suggesting that SMAD3 activation may have a later onset or is masked by high levels of phospho-SMAD3 within control ovaries at this stage. TGFBR1CA was detected using an anti-HA antibody. ACTB was included as internal control. n = 3-4. Each lane represents an independent sample (TIFF 366 kb) [file 12958_2017_312_MOESM2_ESM.tif]

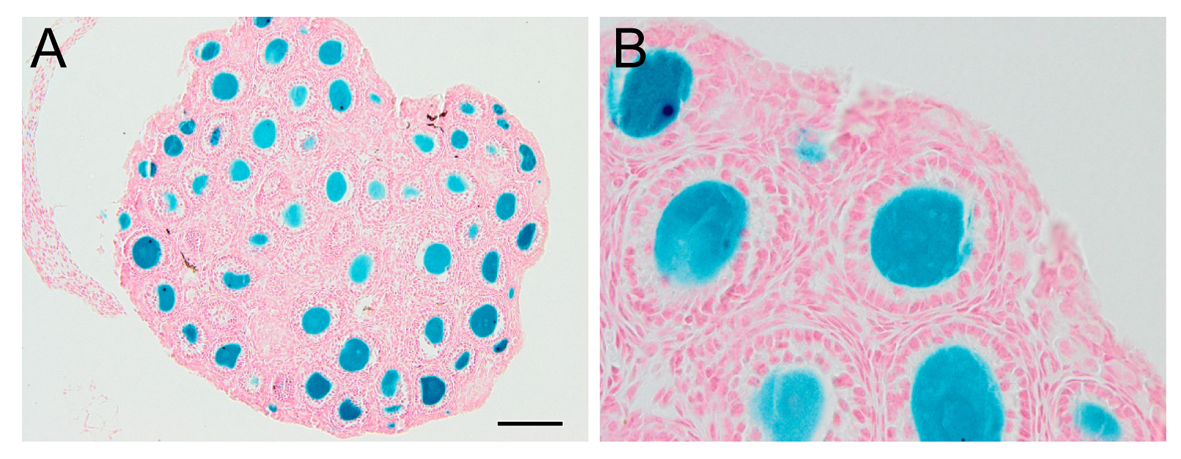

Supplement: Supplementary file 3 — Reporter analysis of Zp3-Cre activity in the ovary. (A and B) X-gal staining of ovaries from Rosa26/Zp3-Cre mice at PD14. Panel (B) is a higher magnification image of panel (A). Results represent staining using 3 independent samples. Scale bar is representatively shown in (A) and equals 25 μm (B) and 100 μm (A) (TIFF 1070 kb) [file 12958_2017_312_MOESM3_ESM.tif]
